# Twice-Daily Scrambler Therapy for a Patient With Spinocerebellar Ataxia Suffering From Refractory Neuropathic Back Pain: A Case Report

**DOI:** 10.7759/cureus.108222

**Published:** 2026-05-04

**Authors:** Pak Hei Tristan Chan, Ka Lai Chu

**Affiliations:** 1 Department of Anesthesiology, Pain Medicine, and Operating Services, United Christian Hospital, Kwun Tong, HKG

**Keywords:** case report, neuropathic pain, sca3, scrambler therapy, spinocerebellar ataxia, type 3

## Abstract

Spinocerebellar ataxia (SCA) is a group of progressive neurodegenerative diseases. These patients often suffer from pain with multiple etiologies. Our patient suffers from distressing neuropathic back pain, which was only marginally alleviated with high-dose conventional antineuropathic agents while suffering from the side effects. A course of scrambler therapy was prescribed, and sustained relief of neuropathic back pain was achieved for at least eight weeks after completion of scrambler therapy. This is the first case of neuropathic pain in SCA to be treated with scrambler therapy, to the best of our knowledge. Compared with usual daily scrambler therapy sessions, our patient received twice-daily sessions. The main takeaways from our case are that 1) scrambler therapy is a potential treatment for neuropathic pain in SCA patients; 2) it can treat neuropathic pain refractory to conventional antineuropathic agents with the added benefit of reducing the requirement as well as side effects associated with conventional antineuropathic agents; and 3) twice-daily sessions are a potential alternative to standard daily sessions with positive logistical implications.

## Introduction

Spinocerebellar ataxia (SCA) is a group of progressive neurodegenerative diseases that are inherited in an autosomal-dominant manner, with SCA type 3 being the most common [1]. As the name suggests, it involves cerebellar degeneration; however, there is also extracerebellar involvement, such as brainstem involvement or peripheral neuropathies, depending on the subtype [2]. Chronic pain is reported to occur in up to 50% of SCA type 3 patients [3]. Although peripheral neuropathy is frequently encountered in SCA type 3 [2], the pain is often multifactorial, usually involving a mechanical component and occasionally a neuropathic pain component [3].

According to the International Association for the Study of Pain, neuropathic pain is the pain caused by "lesion or disease of the somatosensory nervous system." Although there are multiple pharmacological and neuromodulatory therapies, neuropathic pain is difficult to treat, and results are "generally modest" [4].

Scrambler therapy is an FDA-approved treatment for neuropathic pain [5]. It is a noninvasive treatment that uses skin electrodes to produce a variety of nonpainful signals with different waveforms, aiming to replace noxious sensations with nonpainful ones [5]. The artificial, nonpainful signal travels up the same pathway as a pathological endogenous pain signal, thereby remodulating the pain system, and retrains the brain to interpret the previously painful areas as nonpainful areas [5-7]. There are several reports of the success of scrambler therapy for the treatment of chronic pain, particularly those involving peripheral neuropathic pain [5-7].

Here, we present the first reported case of scrambler therapy applied to treatment-resistant neuropathic back pain in a patient with SCA type 3.

## Case presentation

A patient with SCA type 3 was referred to the Kowloon East Cluster Pain Management Centre for severe neuropathic back pain. He first presented to medical services in 2016 with the chief complaints of unsteady gait and repeated falls. Physical examination showed positive cerebellar signs, including horizontal nystagmus, impaired finger-nose test, ataxic gait, and normal power. After extensive workup, including multiple brain imaging and genetic studies, a diagnosis of SCA type 3 was made. His younger brother suffered from less severe symptoms and was confirmed to have SCA type 3 on genetic studies as well. In hindsight, their mother suffered from similar distressing neurological symptoms and passed away before a formal diagnosis was made.

Over the years, the cerebellar symptoms deteriorated, and he became wheelchair-bound about eight years after the diagnosis. There was no sensory symptom in the early days of the disease. In 2020, four years after the onset of cerebellar symptoms, back pain was first documented. It was initially mechanical and attributed to progressive scoliosis. Later, the pain progressed to be predominantly neuropathic, with burning and pinprick sensations. The pain responded poorly to nonsteroidal anti-inflammatory drugs and paracetamol (acetaminophen), while tramadol and pregabalin provided partial relief. The pain led to multiple hospital admissions. In 2022, the first documentation of a neuropathic component of the back pain was described as a "burning sensation." Tramadol was added and initially provided temporary relief. He had his first admission to the hospital for back pain in 2023. The pain was attributed to the progressive scoliosis. Pregabalin was added, with partial improvement of pain. He had several more admissions for lower back pain, which by then had become predominantly burning and pinprick rather than mechanical in nature. The dosages of pain medications, including paracetamol, nonsteroidal anti-inflammatory drugs, and pregabalin, were increased. He was eventually referred to the United Christian Hospital pain clinic in 2025.

He also had psychiatric follow-ups for low mood and sleeping disorders. The psychiatrist treated him with psychiatric drugs such as trazodone and amitriptyline, which happen to have antineuropathic properties.

A course of scrambler therapy was provided following the methodology of the inventor and the distributor, as outlined on scramblertherapy.org [8,9]. The only deviation was the transition to twice-daily sessions starting with the fifth session, in the hope of reducing travel time to and from the hospital. The nature of pain, the pain score at the start, the lowest pain score achieved, the pain score at the end, and the time of sustained pain relief were recorded after each session. A standard course of 10 sessions was initially prescribed, with an additional two sessions added at the end of the 10th session, as complete elimination of pain was not achieved and significant improvements continued to occur, in line with the manufacturer’s recommendation [8]. The painful areas were treated until zero neuropathic pain is achieved, and successful treatment is considered if sustained relief from neuropathic pain is achieved for 48 hours. After completing 12 sessions, there was sustained relief from neuropathic pain for a duration of eight weeks.

Change in pain scores with scrambler therapy

**Figure 1:**
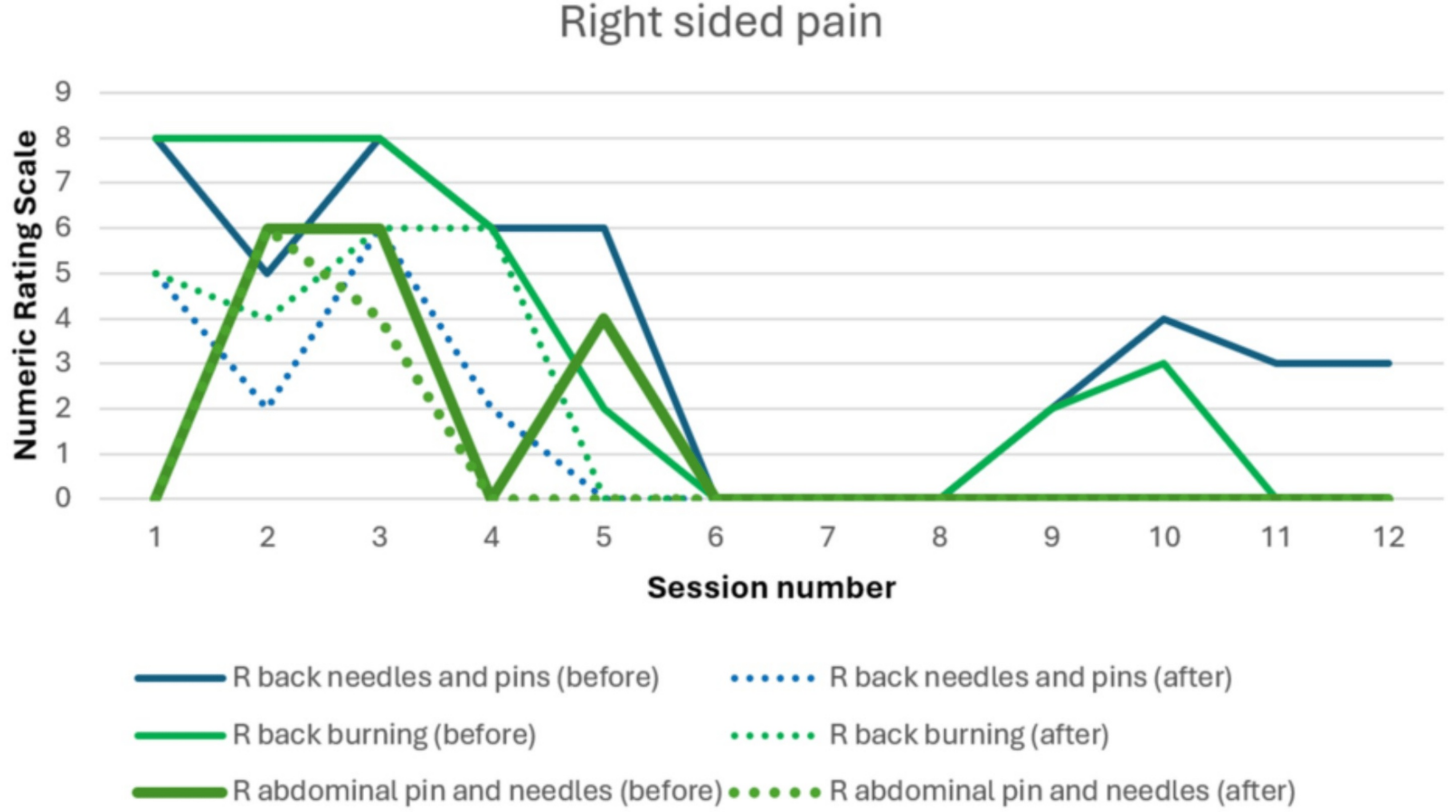
Neuropathic pain on the right side of the body before and after each treatment session

**Figure 2:**
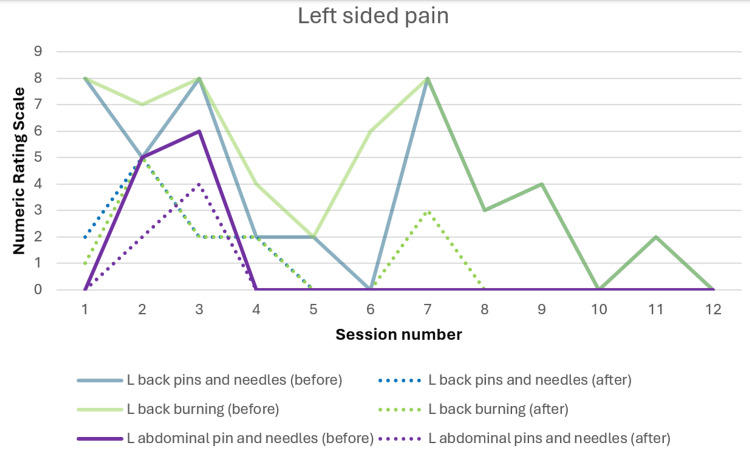
Neuropathic pain on the left side of the body before and after each treatment session.

Figures 1, 2 show the change in neuropathic pain over the course of scrambler therapy. Pain scores responded to the treatment from the first session. The course of pain fluctuates, which is not uncommon, and shows an overall downtrend. There is also development of pain in new areas after treatment, such as pins and needles pain over the abdominal wall, which is known to occur according to the user manual, and the new painful areas were treated as well. The importance of listening to the patient and adjusting the electrode placement is stressed by the manufacturer [9].

**Figure 3:**
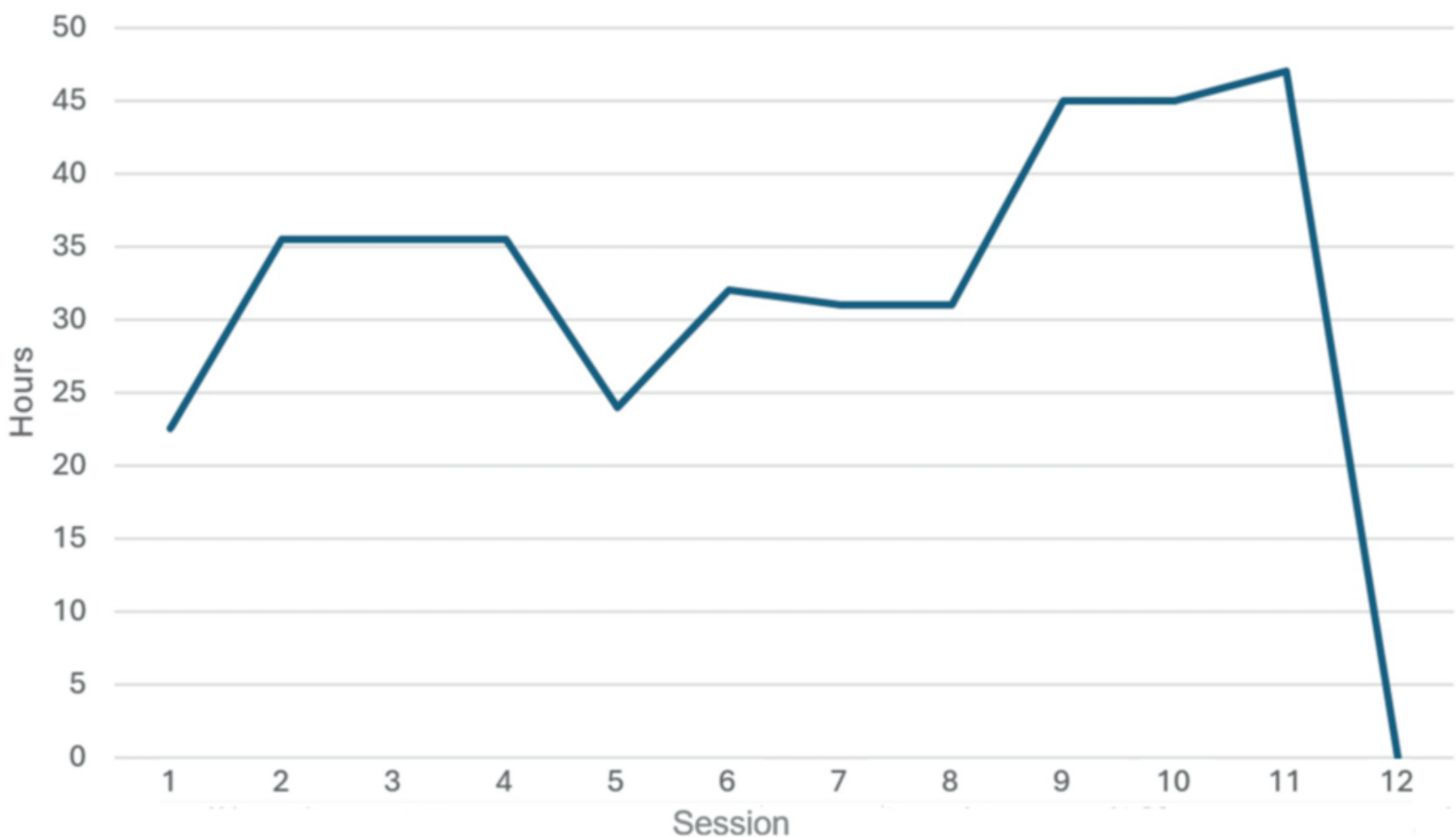
Time to goal of sustained pain relief for 48 hours

The duration of sustained pain relief from neuropathic pain was documented after each session. The temporal relationship between sessions is not fixed; some are separated by a weekend, and daily sessions transitioned to twice-daily sessions starting with the sixth session. Thus, it is more reasonable to present the data as a time difference to our goal of sustained pain relief of 48 hours, as shown in Figure 3.

## Discussion

There has not been any report on the use of scrambler therapy for the treatment of neuropathic pain in patients with spinocerebellar ataxia of any type at the time of writing this case report. Although the cerebellum is the main organ affected, SCA is often not confined to the central nervous system [10]. As peripheral neuropathy is often implicated in SCA3 [10], it is worth considering scrambler therapy, which has had successes in other forms of peripheral neuropathy such as chemotherapy-induced peripheral neuropathy [11].

The causes of back pain in SCA patients are multifactorial, as suggested by Roberts et al. [2] and Soliman et al. [4]. In our patient, pain initially began as mechanical and nociceptive in nature, which is not surprising given the progressive scoliosis. However, as the disease progressed, the neuropathic component became more distressing, to the point that it affected the patient's sleep and mood and required hospital admission.

The patient was already on high doses of nonopioids, antineuropathic agents, and weak opioids, with modest effects on the neuropathic pain while experiencing many of the side effects, namely dizziness, respiratory depression, and autonomic dysfunction. As the disease progresses, some of the side effects of pain medications may eventually become intolerable or cause significant harm to the patient.

The most distressing side effect from medications, according to the patient, was dizziness. Pregabalin, trazodone, and dihydrocodeine are known to cause dizziness, especially when used at high doses, as in our patient. Although dizziness may be a symptom of SCA, the disappearance of dizziness after weaning pregabalin suggests that the medication contributed significantly to dizziness.

Respiratory insufficiency is multifactorial in patients with SCA, and its severity depends on the disease type and progression [12]. There are central and peripheral components of respiratory insufficiency [12], which may compound the respiratory depressive effect of pregabalin.

Many of the antineuropathic agents the patient received can cause autonomic dysfunction [13,14]. One such side effect noted by the patient was urinary retention, which may be caused by trazodone and pregabalin. Like respiratory depression, urinary retention may develop as part of the natural history of SCA, and the pain medications may make the patient more prone to urinary retention.

It is promising that scrambler therapy, being noninvasive and nonpharmacological in nature, is effective in this SCA patient with treatment-resistant neuropathic back pain. By the end of the treatment, there is sustained pain relief for more than eight weeks. Moreover, the patient weaned off pregabalin, with subjective improvement in dizziness. There is also improvement in sleep and mood, allowing room for further titration of trazodone and psychiatric meds.

Intuitively, a shorter time to sustained 48-hour pain relief is “better,” suggesting the patient experiences a longer duration of pain relief. However, this is not apparent in Figure 3, which instead shows a trend away from our goal until the last session. Although the duration of “effective” treatment appears to be shortening, it is important to note that it does not account for the intensity of the pain and, more importantly, that, according to the patient, it never returns to a more painful score than at the start of treatment. Also, we did not record the duration of effect for each location, so Figure 3 shows only the area with the shortest duration of effect, even if all other areas are pain-free. This fluctuation in the duration of treatment effect across space and time is compatible with the underlying theory of scrambler therapy and the neuroplastic nature of pain pathways. Just like humans “learn” at different paces, pain pathways also exhibit this variability. Although the last bit of pain is more “stubborn” and not as distressing to the patient, it is worthwhile to perform more sessions than the standard 10-session therapy if the patient continues to respond to treatment, because, in our patient, once a sustained relief for 48 hours is achieved, it can be sustained for at least another eight weeks.

It is also encouraging to see that scrambler therapy, delivered twice daily, is effective, even though its course developers recommend daily sessions. SCA patients, as in our patient, will eventually have limited mobility. This makes traveling to and from the hospital or clinic difficult. A more intensive schedule, while retaining treatment efficacy, is an attractive future direction and is not limited to use on SCA patients. However, patients have varying requirements for scrambler therapy, and ultimately, the regimen needs to be individualized to balance analgesic and logistical efficacy.

Although this is a rare disease, the demonstration of an effective treatment with minimal side effects for neuropathic pain associated with SCA is important for this group of patients. A previous series has shown that patients with SCA type 3 have an average life expectancy of 20 years from disease onset [15]. Our patient has developed almost intolerable pain after eight years since the initial presentation to medical services. One can only imagine the suffering of living with the pain, as well as the progressive disability, for another 10 years. Furthermore, given the autosomal dominant nature of SCA and that symptoms may occur after reproductive age, as in our patient, it is possible to see more SCA patients in the future. Our patient has fathered two children before the disease onset; they are currently asymptomatic, and after genetic counseling, it has been decided that the children will decide for themselves whether to undergo genetic testing.

SCA is a progressive disease, and peripheral neuropathies will continue to develop. It will not be surprising to see the patient develop pain in new areas. It is also known that relapses of neuropathic pain can occur after initial successful scrambler therapy [6]. Fortunately, there is no limit to the number of scrambler therapies, according to provider instructions, if the patient continues to respond to treatment [8,9]. There have been reports of booster sessions, with as few as one or two, to reestablish the previously achieved pain-free [6]. We will continue to follow up on the patient’s pain condition to see if neuropathic pain recurs and provide timely support.

## Conclusions

Spinocerebellar ataxia is a rare and diverse group of neurodegenerative diseases. Pain in SCA patients may involve both mechanical and neuropathic components. It is important to keep in mind that this is only a single case report, and the effectiveness and scheduling frequency may not be generalizable to other SCA patients; further trials may be needed. Our case has shown that scrambler therapy is a potential treatment for neuropathic pain in these patients. In our patient, sustained remission from neuropathic pain was achieved for at least eight weeks, allowing weaning of systemic medications and, more importantly, the side effects of the medications. Furthermore, we have demonstrated that a twice-daily schedule, rather than the recommended once-daily schedule, has the potential to treat neuropathic pain effectively as well.
